# Multipotent Capacity of Immortalized Human Bronchial Epithelial Cells

**DOI:** 10.1371/journal.pone.0022023

**Published:** 2011-07-07

**Authors:** Oliver Delgado, Aadil A. Kaisani, Monica Spinola, Xian-Jin Xie, Kimberly G. Batten, John D. Minna, Woodring E. Wright, Jerry W. Shay

**Affiliations:** 1 Department of Cell Biology, University of Texas Southwestern Medical Center at Dallas, Dallas, Texas, United States of America; 2 Hamon Center for Therapeutic Oncology Research, University of Texas Southwestern Medical Center at Dallas, Dallas, Texas, United States of America; 3 Department of Clinical Sciences, University of Texas Southwestern Medical Center at Dallas, Dallas Texas, United States of America; Kings College London, United Kingdom

## Abstract

While the adult murine lung utilizes multiple compartmentally restricted progenitor cells during homeostasis and repair, much less is known about the progenitor cells from the human lung. Translating the murine stem cell model to humans is hindered by anatomical differences between species. Here we show that human bronchial epithelial cells (HBECs) display characteristics of multipotent stem cells of the lung. These HBECs express markers indicative of several epithelial types of the adult lung when experimentally tested in cell culture. When cultured in three different three-dimensional (3D) systems, subtle changes in the microenvironment result in unique responses including the ability of HBECs to differentiate into multiple central and peripheral lung cell types. These new findings indicate that the adult human lung contains a multipotent progenitor cell whose differentiation potential is primarily dictated by the microenvironment. The HBEC system is not only important in understanding mechanisms for specific cell lineage differentiation, but also for examining changes that correlate with human lung diseases including lung cancer.

## Introduction

Repair and regeneration of the adult lung is critical in order to maintain its integrity and functional capacity. Studies on lung development and postnatal repair utilizing murine models have provided valuable insights into both lung homeostasis and regeneration. These studies have demonstrated that the adult mouse lung epithelium is relatively quiescent and does not adhere to the classic stem cell model [1]. Instead, the lung appears to conform to a maintenance scheme similar to that of other tissues with slow turnover rates, such as the pancreas [2], [3].

During normal tissue homeostasis, “abundant” facultative progenitor cells situated throughout the lung epithelium mediate any necessary maintenance. These facultative progenitor cells, Clara cells and Type II pneumocytes, are quiescent and function as differentiated cells of the mature lung epithelium, but retain the capacity to self-renew and differentiate into other lung epithelial cell types during normal homeostasis [1], [4], [5]. When the lung tissue is injured, putative lung stem cells, which reside in distinct niches within the adult murine lung epithelium, are induced to proliferate and assist in tissue regeneration. Currently, only basal cells and a subset of Clara cell secretory protein (CCSP) expressing cells, termed variant CCSP-expressing cells (vCE), retain this higher potency in the murine lung [1], [6], [7], [8], [9].

This organization of adult murine lung stem cell hierarchy is efficient, but limited *in vivo* due to the restricted potential of these cells such that they can only regenerate epithelial cells within their resident anatomical compartment. Basal, Clara, or vCE cells only regenerate the airways while the Type II pneumocytes only regenerate the alveoli [5], [6], [9], [10]. However, during early lung development there is multipotent embryonic progenitor cell type that is capable of differentiating into all epithelial cell types of the lung [11], [12]. Recent *in vitro* evidence has additionally suggested that multipotent progenitor cells exist within the adult murine lung and are postulated to give rise to all of the stem/progenitor cells described above [13].

While much has been elucidated concerning maintenance of the adult murine lung, many details remain uncertain thus impairing the direct translation of this model to humans. Basal cells are only found within the trachea of mice while they are present throughout the human airways [14], [15]. Inversely, Clara cells are predominantly confined to the most distal bronchiole airways in humans, but are found throughout the murine airways [10], [16]. Further, vCE cells have only been shown in mice and there is no evidence that these cells exist in humans. One alteration to this regeneration model that would accommodate the incompatibility caused by these differences is if human basal cells function analogously to the murine Clara or vCE cells during homeostasis and repair. A method for isolation of a purified population of basal cells from the human trachea has recently been described, however, these cells did not demonstrate the capacity to differentiate into Clara or goblet cells under their *in vitro* conditions [6]. Whether these results are indicative of the potential of all human basal cells in not known.

In this study, we evaluated the *in vitro* potential of human bronchial epithelial cells (HBECs). We demonstrate that these cells are not restricted *in vitro* from differentiating into epithelial cell types of different anatomical compartments of the lung, including Type II pneumocytes. We report that subtle changes in the microenvironment result in unique responses including the ability of HBECs to differentiate into multiple central and peripheral lung cell types. These studies demonstrate that differences exist in stem cell hierarchy between humans and mice and that the microenvironment impacts the differentiation program of lung stem cells.

## Results

### Response of Immortalized HBECs in Several Three-Dimensional (3D) Organotypic Culture Models

**Figure 1:**
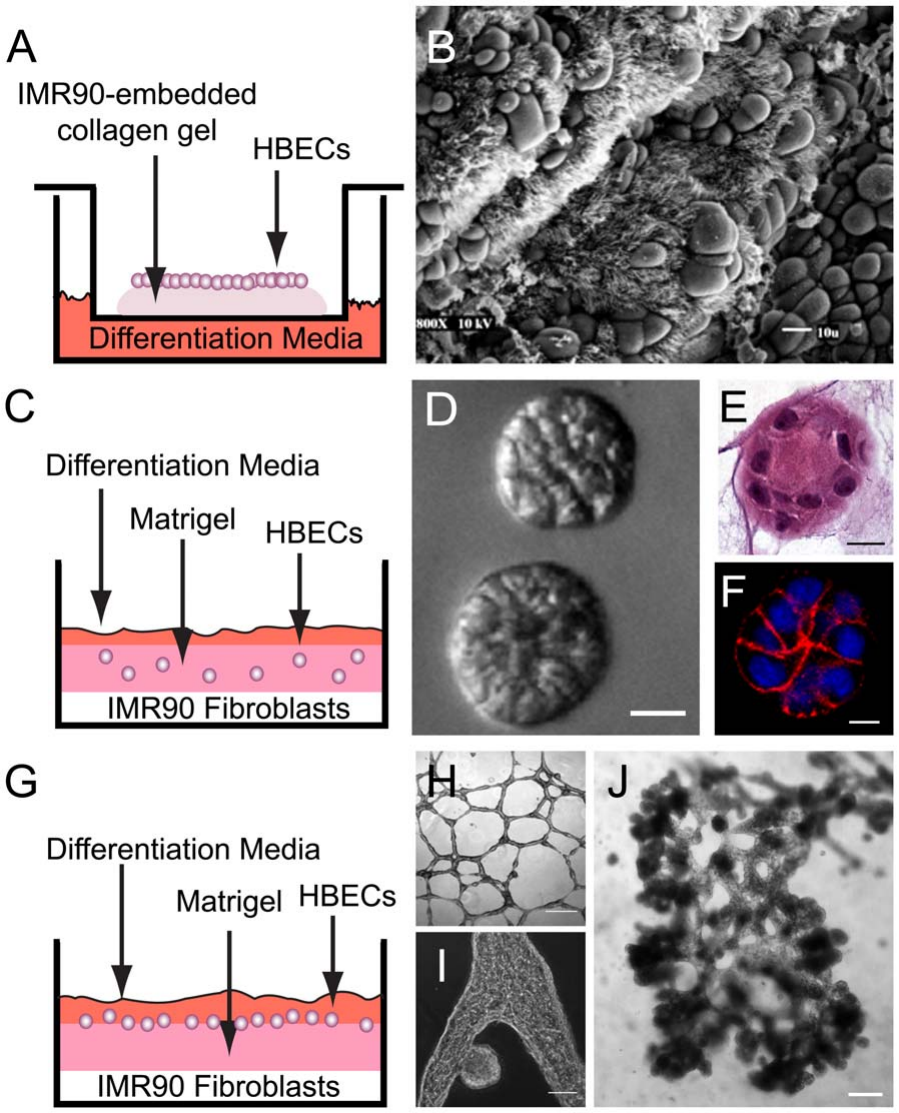
HBEC3 KTs Respond Distinctly to Cues from Microenvironment. HBEC3 KTs were seeded into three different 3D culture models (Figs A, C, and G) and overall response monitored. Culture of the HBEC3 KT cells atop of an IMR-90-embedded collagen type I matrix (Fig A) results in the differentiation into ciliated and/or goblet cells. Scanning electron micrograph (EM) of apical surface of HBEC3 KTs after 3 weeks at an air-liquid interface (Fig B; scale bar 10 µm). Embedding single HBEC3 KT cells within Matrigel™ in the presense of IMR90 fibroblasts (Fig C) results in the formation of organized cyst-like structures comprised of cuboidal cells. Phase contrast image, H&E section, and immunofluorescence staining for E-cadherin (red) in HBEC3 KT cyst-like structures after 5 days of culture (Figs D, E, and F respectively) (scale bar 20 µm; DAPI (blue)). Alternatively, seeding of HBEC3 KT cells on top of Matrigel™ in the presence of IMR90 fibroblasts (Fig G) results in the aggregation into tubular structures from which bud structures emerge over time. Phase contrast images of HBEC3 KTs 24 hours after plating on Matrigel™ (Fig H; scale bar 100 µm), of budding structure (Fig I; scale bar 20 µm), and after 5 days in culture (Fig J; scale bar 200 µm). Results are representative of >20 independent experiments.

Previously, our lab demonstrated that HBEC3s, which were obtained from a central lung bronchiole, could be immortalized with the ectopic expression of cyclin-dependent kinase 4 (CDK4; K) and human telomerase reverse transcriptase (hTERT; T). These HBEC3 KT cells were further shown by several methods to retain a stable normal phenotype thus permitting the analysis of an isogenic population of normal lung epithelial cells without the confounding influence of any senescence-associated alterations or oncogenic transformation [17], [18], [19]. Early characterization of these HBEC3 KT cells revealed that these cells retain expression of the epithelial stem/progenitor cell marker tumor protein p63 (p63) and the capacity to differentiate into both ciliated and goblet cells when seeded on top of a contracted fibroblast matrix and then raised to an air-liquid interface for four weeks (Figures 1A and 1B) [18], [19]. These results suggest that the HBEC3 KT cells are akin to basal cells under these culture conditions [1], [6], [20]. However, these earlier analyses did not determine whether the HBEC3 KT cells also have the capacity to differentiate into Clara cells as has been demonstrated for the murine basal cells *in vivo* [1], [6], [9].

To further evaluate the potential of the HBEC3 KTs and determine whether cues from the microenvironment influences their ability to differentiate into other lung epithelial cell types, we cultured the HBEC3 KTs both within and atop of reconstituted basement membrane (Matrigel™) (3D) (Figures 1C and 1G). When co-cultured in Matrigel™ below a monolayer of IMR90 fetal lung fibroblasts, HBEC3 KT cells formed spheroid cyst-like colonies approximately 50 µm in diameter within five days of culture (Figure 1D and Figures S1A and S1B). Within these cyst-like structures, HBEC3 KTs initially retained a cuboidal morphology (Figure 1E) and formed well-defined adhesion junctions with neighboring cells (Figure 1F). HBEC3 KT cells did not proliferate in the absence of fibroblasts (Figures S1A and S1B).

When the HBEC3 KT cells were seeded on of Matrigel™, these cells self-aggregated into tubule structures (Figure 1H) similar to endothelial cell organotypic cultures [21]. Small bud-like structures became apparent along the sides of these tubules by the third day of culture (Figure 1I). These budding structures continue to increase in number and size by 10 days (Figure 1J). These observations provide morphological evidence that the HBEC3 KT cells retain the capacity to produce structures resembling those in the periphery of the lung such as branched alveoli.

### Multipotent Stem Cell Maintenance Genes Are Differentially Regulated and Dependent on Culture Conditions in HBEC3 KTs

**Figure 2:**
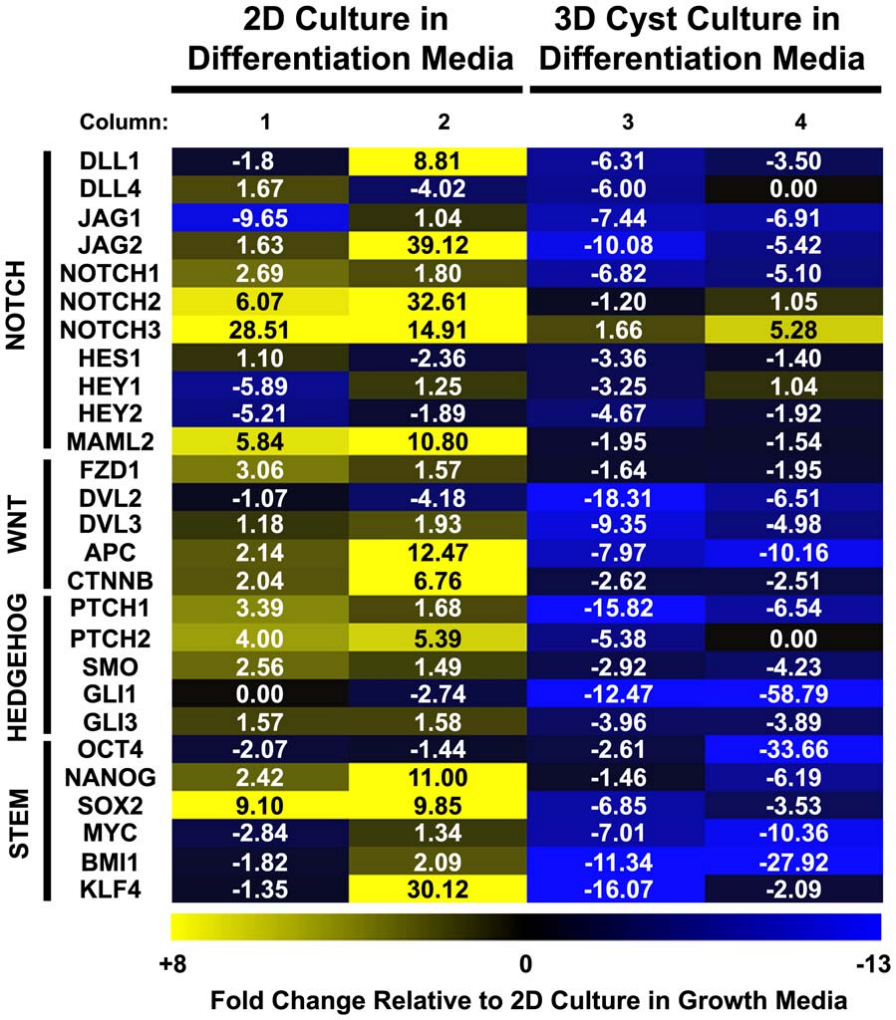
HBEC3 KT Cells Differentially Regulate Genes Associated with Progenitor Maintenance. Quantitative RT-PCR analysis of several genes within pathways critical for progenitor cell maintenance in HBEC3 KT cells five days after varying culture conditions. Transition of HBEC3 KT cells from standard growth media to differentiation media while cultured in 2D results in the significant upregulation of many genes within progenitor-associated pathways (Columns 1 and 2) relative to HBEC3 KT cells cultured within Matrigel™ with differentiation media and IMR90 fibroblast growth stimulus (Columns 3 and 4) (*P* = 0.00185). Results are from two independent experiments (Columns 1 and 3 and 2 and 4, respectively). Experimental samples normalized to gene expression in HBEC3 KT cells coincidentally maintained in growth media.

Progenitor cell activation, either through injury or during normal homeostasis, requires the proliferation of these cells in addition to the integration of extrinsic cues from their microenvironment for proper specification and differentiation [22]. Modulation of signaling pathways, such as Notch, Wnt, and Hedgehog, is known to be critical for both progenitor cell maintenance and differentiation in several tissues including the lung [12], [22], [23]. The distinct response of the HBEC3 KT cells to each of the 3D models tested suggested that these cells are particularly sensitive to subtle changes in their environment and thus these pathways may play a role in the HBEC3 KT response. In order to determine whether genes within these progenitor-associated pathways are expressed in the HBEC3 KT cells and whether this expression is influenced by environmental factors present in cell culture, such as media composition or cellular substratum, quantitative real-time PCR was performed on the HBEC3 KT cells upon varying these environmental factors. All of the 3D models tested require culturing the HBEC3 KT cells in media formulated to promote their differentiation [18], [24]. The HBEC3 KT cells were thus transitioned from standard growth to differentiation media and cultured for five days as a 2D monolayer to determine the influence of the culture medium on the expression of multiple progenitor-associated genes. Five days after transitioning the HBEC3 KT cells from standard growth to differentiation media several genes within these progenitor-associated pathways were upregulated in the HBEC3 KTs cultured in differentiation media when compared to HBEC3 KTs maintained in growth media (Figure 2). SRY-box 2 (Sox2), which has roles in both the developing and adult lung, was also upregulated (Figure 2) [25]. Interestingly, Sox2 is transiently upregulated *in vivo* in the adult bronchiolar epithelium after injury and is required for proper maintenance and differentiation [26], [27]. These results indicate that, by altering the media composition in 2D culture, the HBEC3 KT cells are capable of engaging the appropriate mechanisms to respond to extrinsic signals that modulate their differentiation lineage.

To determine whether the 3D culture microenvironment additionally impacts the expression of these genes, the HBEC3 KT cells were cultured within Matrigel™ into cyst-like structures for five days as described above. Although the HBEC3 KTs were cultured with the same differentiation media assayed in 2D, almost all of the genes analyzed are significantly downregulated (*P* = 0.00185) when compared to HBEC3 KTs cultured in 2D with differentiation media and normalized to the expression in HBEC3 KTs maintained in standard growth media (Figure 2).

### Analysis of Differentiated Epithelial Markers in HBEC3 KTs Cultured as a Two-Dimensional (2D) Monolayer

**Figure 3:**
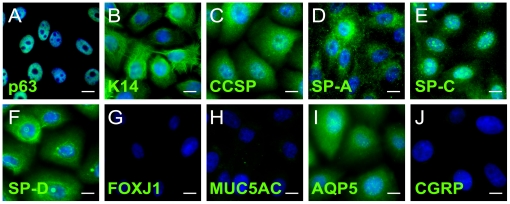
Immortalized HBEC3s Simultaneously Express Markers of Multiple Cell Types of the Lung. Immunofluorescence staining of HBEC3 KTs cultured as 2D monolayer in differentiation media for two days demonstrates capacity of HBEC3 KTs to express markers from multiple lung cell types while actively proliferating. (A) p63. (B) K14. (C) CCSP. (D) SP-A. (E) SP-C. (F) SP-D. (G) FOXJ1. (H) MUC5AC. (I) AQP5. (J) CGRP. DAPI, blue. Scale bars 10 µm. Results are representative of five independent experiments.

Every mature epithelial cell type in the adult lung, including the putative lung stem/progenitor cells, can be identified by the expression of a unique differentiation marker or the combination of a few markers. Embryonic multipotent progenitor cells of the developing murine lung, however, simultaneously express markers indicative of several differentiated epithelial cell types suggesting that the expression of differentiation markers is unrestricted in activated multipotent progenitors [28]. In order to determine the HBEC3 KTs marker profile, we performed immunofluorescence staining on HBEC3 KTs in 2D monolayer with a panel of these markers 48 hours after transitioning these cells from standard growth to differentiation media as was shown to lead to the upregulation of progenitor-associated genes. Consistent with previous results, the basal cell marker p63 was expressed (Figure 3A) [19] in addition to another marker of basal cells, Keratin 14 (K14) (Figure 3B) [6], [9]. CCSP, a marker for Clara cells that has limited expression in Type II pneumocytes [10], was also uniformly expressed in the HBEC3 KT cells (Figure 3C). Intriguingly, we found that the HBEC3 KTs were positive for all three of the surfactant proteins tested, surfactant protein-A, -C, and –D (SP-A, SP-C, and SP-D) (Figures 3D–3F, respectively). SP-A and SP-D are expressed in both Type II pneumocytes and Clara cells [29], [30] while the expression of SP-C is restricted to Type II pneumocytes [31], [32]. At the same time, HBEC3 KT cells were positive for aquaporin 5 (AQP5), a marker for Type I pneumocytes that is potentially expressed in many other cell types of the lung including basal cells (Figure 3I) [33]. Staining for markers of mature ciliated, goblet, and neuroendocrine cells (forkhead box J1 (FOXJ1), mucin 5AC (Muc5ac), and calcitonin gene related peptide (CGRP)) (Figures 3G, 3H, and 3J, respectively) [34], [35], [36] and secondary only controls (data not shown) were all negative under these conditions. As expected, thyroid transcription factor 1 (TTF1), the regulator of several of the peripheral lung epithelial markers tested, [37] was also detected in the HBEC3 KT cell line but at variable levels (Figure S2A–S2D).

### Potential for Alveolar Differentiation Retained in HBEC3 KT Cells

**Figure 4:**
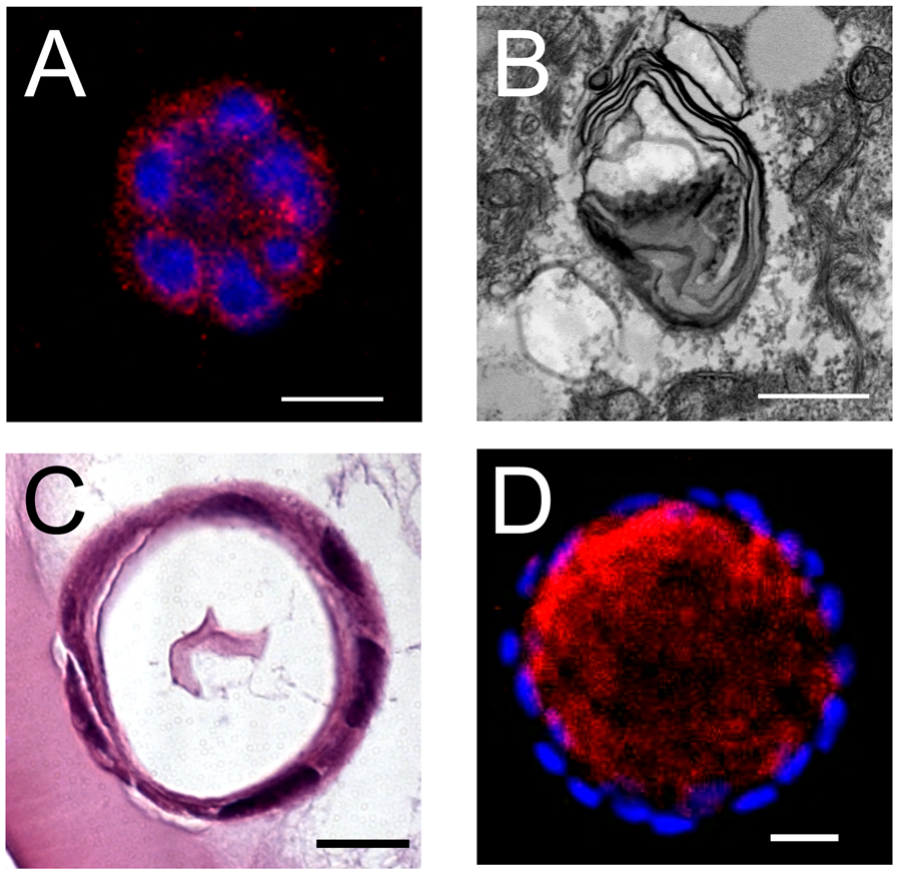
Lamellar Bodies Detected in Subsets of HBEC3 KT Cyst-like Structures after Extended Culture. HBEC3 KT cells were cultured within Matrigel™ for extended periods of time and the potential for differentiation into distal lung cell type monitored. After 7 days in culture, HBEC3 KT cells retain SP-A expression in punctate foci throughout the cytoplasm with subsets of structures containing lamellar bodies. Immunofluorescence staining of SP-A (red) (Fig A; DAPI (blue); scale bar 20 µm) and transmission EM of lamellar bodies (Fig B; scale bar 0.5 µm) in HBEC3 KT cyst-like structures after 7 days of culture. Further culture of HBEC3 KT cells within Matrigel™ results in cyst-like structures comprised of squamous cells surrounding an luminal space in which SP-A is detected. Representative H&E image (Fig C; scale bar 20 µm) and immunofluorescence staining of SP-A (red) (Fig D; DAPI (blue); scale bar 30 µm )in HBEC3 KT cyst-like structures after 9 days of culture. Results are representative of three independent experiments.

The HBEC3 KT expression profile, SP-C in particular, suggests that these cells may retain the capacity to differentiate into lung alveolar epithelial cells. Lamellar bodies are lipid storage organelles that can be detected throughout the cytoplasm of Type II pneumocytes by electron microscopy [32]. Transmission electron microscopy on HBEC3 KT cyst-like structures after 7 days of culture detected lamellar bodies within individual HBEC3 KT cells (Figure 4B). Generally, these lamellar bodies were surrounded by lipid filled vesicles. SP-A is commonly associated with lamellar bodies and was present in punctate foci in the cyst-like structures at this time (Figure 4A). When cultured for extended periods of time, these structures develop into cysts comprised of squamous cells surrounding a clear lumen (Figure 4C). SP-A was observed to localize within the lumens of these structures (Figure 4D).

## Discussion

Currently, it is unknown how stem cells of the murine or human lung are regulated. In certain systems, epigenetic changes permanently alter the transcriptional program of a stem cell or differentiating cell [38], [39]. The microenvironment in which a cell resides, such as the stem cell niche, has also been shown to impact the potential of a cell [39], [40]. Comparison of the different lung compartments in which the murine stem cells reside suggests that the restricted potency *in vivo* could be determined, at least in part, by their microenvironment. It is reasonable to assume that by placing cells in culture any environmental restrictions would be modified or eliminated, thus permitting the inherent potential of the cells to be assessed. Our results demonstrate that the HBEC3 KT cell line retains a multipotent capacity *in vitro*, which supports the concept that the local microenvironment may influence the potency of the lung stem cells *in vivo*.

Several reports have described the formation of spheroid structures with defined populations of lung epithelial cells when cultured within Matrigel™ allowing for more direct comparisons to *in vivo* models [6], [13], [41]. Isolated tracheal murine and human basal cells cultured in a diluted Matrigel™ suspension, with identical medium and in the absence of a fibroblast-mediated growth stimulus, formed “tracheospheres”. These isolated basal cells, however, were not shown to be capable of differentiation into Clara or goblet cells under these conditions and are therefore restricted in their differentiation potential compared to basal cells *in vivo* [1], [6], [9]. Whether this restriction is intrinsic or extrinsic is not known.

Putative multipotent progenitor cells isolated from the adult murine lung also form spheroid structures from individual cells, but only when co-cultured with α-smooth muscle actin positive (αSMA^pos^) mesenchymal cells in the same diluted Matrigel™ suspension. While subsets of these colonies contain cells that express markers of both airway and alveolar lineages, no individual cells were shown to express markers of both compartments [13]. This suggests that the surrounding αSMA^pos^ cells are directly influencing the differential expression observed. Type II pneumocytes are also capable of forming cyst-like structure, although, through cellular aggregation and remain only as functional Type II cells [41]. We are unaware of any reports describing the formation of budding tubules through the aggregation of lung epithelial cells.

Previous characterization of the HBEC3 KTs suggested that these cells resembled basal cells *in vitro* [18], [19]. This possibility was bolstered by the fact that, if the basal cells retain the highest level of potency in the human bronchiole, then they would be the most competent cells to emerge from a lung tissue explant and thus be immortalized. Simultaneous expression of both basal and Clara cell markers suggests that at the time of analysis the HBEC3 KT cells may be in transition between these cell types. There is no evidence that a Clara cell may become a basal cell and thus the directionality of this transition would be predicted to be from a basal cell to a Clara cell. However, the co-expression of SP-C does not correlate with this possibility as it is only expressed in Type II pneumocytes [31], [32].

Multipotent embryonic progenitors of the developing mouse lung have been characterized to co-express differentiation markers for multiple cell types of the lung and give rise to all lung epithelial lineages including neuroendocrine (NE) cells [11], [28]. The simultaneous expression of differentiation markers ascribed to multiple cell types, including all of the putative adult lung stem/progenitor cells, suggests that the HBEC3 KTs resemble multipotent embryonic progenitors in this context. Comparative gene expression analysis of HBEC3 KTs cultured in 2D with differentiation media further suggests that these cells retain similarity to multipotent embryonic progenitors, such as the expression of ID2 (data not shown) [42]. We do not have any evidence to indicate that the HBEC3 KT cells are capable of expressing any markers associated with NE cells, which thus creates a potential distinction between these cells and the putative murine multipotent progenitor cells.

Further, our 2D analyses revealed that the transition of the HBEC3 KT cells from growth to differentiation media is sufficient to induce the upregulation of genes within various stem cell maintenance pathways suggesting that these cells are poised to respond to environmental cues provided. However, whether the temporospatial activation of these pathways prior to or throughout the differentiation process is capable of refining the lineage specification of the cells is not known. Application of various extracellular signals known to be present during the various stages of lung development could further illuminate the true potential the HBEC3 KT cell line.

**Figure 5:**
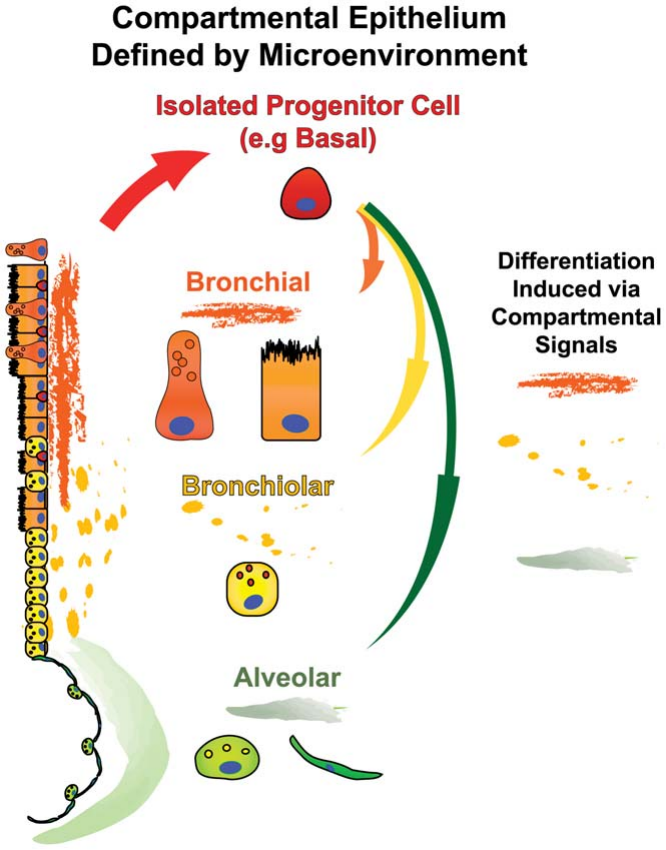
Differentiation of Multipotent Progenitors of Adult Human Lung Dictated by the Microenvironment. Cues provided by the microenvironment encompassing each of the anatomical compartments of the adult human lung provide cues that direct the differentiation of activated multipotent progenitor cells *in vivo*. Differentiation of isolated progenitor cells *in vitro* is thus largely influenced by the extrinsic signals provided.

Overall, our results characterize a novel multipotent capacity of the HBEC3 KT cell line *in vitro*. As these cells were derived from the central airways of an adult lung and display characteristics of a basal cell, we suggest that the HBEC3 KT cells are derived from a basal cell. Maintenance of an actively proliferating population places these cells in the context of a lung epithelial injury *in vivo*. Signals provided by the microenvironment in which these cells are placed when differentiation is induced may therefore dictate the resultant differentiated cell types (Figure 5). Analysis of several known environmental cues from each compartment such as rigidity of substrate, endothelial crosstalk, or activation of progenitor maintenance signaling pathways may provide useful insight into the process of specification. Further, determining how the lung environment regulates the function of its progenitor cells would translate into a better understanding of several lung disorders.

## Materials and Methods

### Cell Culture

HBEC3 cells [19] were cultured in Keratinocyte-SFM containing 50 µg/mL bovine pituitary extract and 5 ng/mL epidermal growth factor (Gibco) at 37°C in 5% CO_2_ on porcine gelatin-coated tissue dishes (Sigma Aldrich) as described previously with the establishment of the HBEC3 cell line [19]. For 2D studies, HBECs were cultured in complete ALI media [24]. IMR90 fibroblasts were cultured in 4∶1 mixture of Dulbecco's Minimal Essential Medium and Medium 199 (Thermo Scientific) containing 10% Cosmic Calf Serum (Thermo Scientific) at 37°C in 2% CO_2_.

### Immunofluorescence Staining

For 2D analyses, HBECs were seeded into chamber slides (Thermo Scientific). After 48 hrs, cells were washed twice with cold PBS and fixed at RT for 10 min (cold methanol for cytoskeletal-associated proteins and 4% paraformaldehyde for all other proteins). Following three washes in PBS, cells were permeabilized and blocked at RT for 30 min with 10% goat serum albumin (Zymed) in PBS containing 0.1% bovine serum albumin (Sigma Aldrich) and 0.1% Triton X-100 (Sigma Aldrich). Cells were rinsed with PBS and incubated with primary antibodies at a dilution of 1∶200 in a humidified chamber at 4°C overnight (rabbit polyclonal anti-p63 (Santa Cruz; sc-8343), mouse monoclonal anti-K14 (Thermo Scientific; Cat. #MS-115-P), rabbit polyclonal anti-CCSP (Santa Cruz; sc-25554), rabbit polyclonal anti-SP-A (gift from Dr. Carole Mendelson, Ph.D., University of Texas Southwestern Medical Center, Dallas, TX [43]), rabbit polyclonal anti-proSP-C (Chemicon; AB3786), mouse monoclonal anti-SP-D (gift from Dr. Jens Madsen, Ph.D., University of Southampton, Southampton, U.K. [30]), mouse monoclonal anti-CGRP (Santa Cruz; sc-57053), mouse monoclonal anti-MUC5AC (Abcam; ab24070), rabbit polyclonal anti-AQP5 (Santa Cruz; sc-28628), mouse monoclonal anti-FOXJ1 (Santa Cruz; sc-53139), and mouse monoclonal anti-TTF1 (Santa Cruz; sc-25331). After three washes in PBS, cells were incubated at RT with secondary antibodies at a dilution of 1∶400 (Alexa Fluor 488 goat anti-rabbit (Invitrogen; A-11008) or Alexa Fluor 488 goat anti-mouse (Invitrogen; A-11001) in the dark for 45 min. Cells were then washed three times with PBS and mounted with VECTASHIELD® Mounting Medium with DAPI (Vector Labs), coverslipped, and imaged at RT on Axiovert 200 M fluorescence microscope with oil objectives EC Plan-Neofluar 40× (NA 1.3) and Apochromat 100× (NA 1.4) (Zeiss). Images were captured with AxioCam MRm camera and AxioVision 4 software (Zeiss). For 3D cyst structures, cultures were washed with PBS and then extracted with Cell Recovery Solution (BD Biosciences) per the manufacturers protocol. Extracted cysts were seeded into chamber slides, allowed to adhere for 4 h, and then fixed and stained according to protocol described above (rabbit monoclonal anti-E-Cadherin (Abcam; ab40772). Imaging was performed on Leica TCS SP5 confocal microscope with HCX PL APO 40× (NA 1.25) and HCX PL APO 63× (NA 1.4) oil objectives using LAS AF software (Leica). lmages were processed with ImageJ and Adobe Photoshop CS2 (Adobe Systems, Inc.).

### 3D Organotypic Culture

Construction and culture of HBECs atop contracted collagen gels was performed as previously described [18]. Culture of HBEC cyst-like structures was performed as described for mammary epithelial cells by Lee *et al.* [44] with following modifications. IMR90 fibroblasts were seeded into a 24-well plate (BD Falcon) in ALI media 48 hrs prior to seeding HBECs. HBEC cells were sub-cultured and re-suspended in ALI media. Cell suspension was diluted 1∶10 in pre-thawed cold Growth Factor Reduced Matrigel™ Phenol-Red Free (BD Biosciences) and 200 µL/well of suspension was overlaid fibroblasts. Matrigel™ cultures were allowed to gel for 30 min at 37°C and then submerged in ALI media. For experiments requiring isolation of cyst-like structures, Matrigel™ cultures were plated in 24-well transwells (Corning) and then transwells were placed atop IMR90 fibroblasts. For HBEC tubule formation, 150 µL pre-thawed cold Growth Factor Reduced Matrigel™ Phenol-Red Free was layered on IMR90 fibroblasts and allowed to gel for 15 min at 37°C. 200,000 HBECs in ALI media were seeded on top of Matrigel™. Cultures were grown at 37°C in 5% CO_2_.

### Electron Microscopy and Hematoxylin and Eosin

Structures were fixed (4% paraformaldehyde for H&E and 2.5% glutaraldehyde (in 0.1 M cacodylate solution) for EM), embedded in 2% agarose, and processed according to standard procedures.

### Quantitative Real-Time PCR

HBEC3 KT cells (6,700 cells/ cm^2^) were cultured for five days with either Keratinocyte-SFM with supplements or complete ALI media in 2D or 200,000 HBEC3 KTs, dispersed as single cells, were cultured within Matrigel™ in transwell supports suspended above fibroblast feeder layer for five days as described above. Total RNA was isolated using RNeasy Plus Mini Kit (Qiagen) per manufacturer's protocol from three plates per growth condition, pooled together for analysis in each experiment. 1 µg of RNA per sample was reverse-transcribed with iScript cDNA Synthesis Kit (Bio-Rad). cDNA from each of three culture conditions were loaded into single 96-well plate and mRNA expression was analyzed utilizing gene-specific TaqMan® probes (Applied Biosystems; Table S1) in triplicate with GAPDH as housekeeping control. The comparative C_T_ method (Applied Biosystems) was used to calculate relative expression values. Expression was normalized to HBEC3 KT samples cultured 2D in Keratinocyte-SFM media. Statistical analysis of expression differences between 2D and 3D with Bonferroni test; P-value cutoff = 0.05).

### RT-PCR

Total RNA was isolated as described above and reverse-transcribed with SuperScript First-Strand Synthesis System (Invitrogen). Intron-spanning primers were utilized for mRNA expression analysis (*TTF1* - Fwd primer – GCCGTACCAGGACACCATG; Rev primer – TTGCTCACGTCCCCCAGCG; *GAPDH* - Fwd primer –CCCATCACCATCTTCCAGGAG; Rev primer - CTTCTCCATGGTGGTGAAGACG).

## Supporting Information

Figure S1 **HBEC3 KT Cells Form Cyst-like Structures when Co-cultured with IMR90 Fetal Lung Fibroblasts.** HBEC3 KT cells were cultured within Matrigel™ with or without IMR90 fetal lung fibroblasts and monitored for up to twelve days. Representative phase contrast images (Fig A) and quantification of HBEC3 KT structure diameter of HBEC3 KT cells grown in Matrigel™ over the course of the assay (Fig B). Scale bars 20 µm. Error bars represent SD from three independent experiments.
(TIF)

Figure S2 **TTF1 Expression in HBEC3 KT Cell Line.** (A) RT-PCR analysis of TTF-1 in HBEC3 KTs cultured under differentiation conditions in 2D or within Matrigel™ compared to human fetal Type II pneumocytes and IMR90 fibroblasts. (B–D) Immunofluorescence staining of TTF-1 in HBEC3 KT cells 2 days (B), 5 days (C), and 7 days (D) after transition from logarithmic to differentiation conditions in 2D. Scale bars 20 µm.
(TIF)

Table S1 **TaqMan® Gene Expression Assay IDs.** TaqMan® Gene Expression Assay IDs and for expression analysis reported in Figure 2.
(DOC)
