# Supplementary figures and images for: Multipotent Capacity of Immortalized Human Bronchial Epithelial Cells
Source: PLoS One. 2011 Jul 7;6(7):e22023. doi: 10.1371/journal.pone.0022023 (PMC3131301; doi:10.1371/journal.pone.0022023)

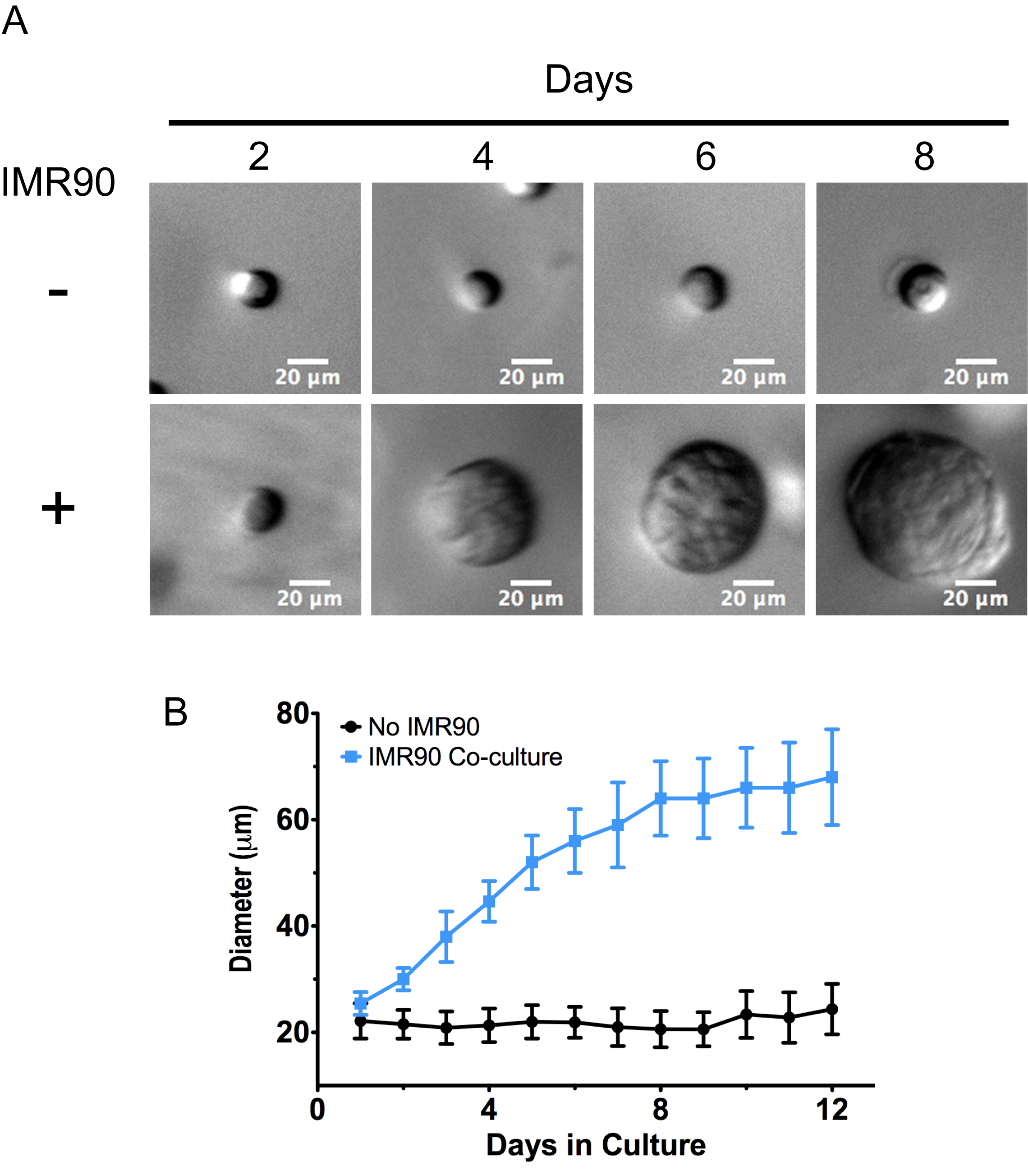

Supplement: Figure S1 — HBEC3 KT Cells Form Cyst-like Structures when Co-cultured with IMR90 Fetal Lung Fibroblasts. HBEC3 KT cells were cultured within Matrigel™ with or without IMR90 fetal lung fibroblasts and monitored for up to twelve days. Representative phase contrast images (Fig A) and quantification of HBEC3 KT structure diameter of HBEC3 KT cells grown in Matrigel™ over the course of the assay (Fig B). Scale bars 20 µm. Error bars represent SD from three independent experiments. (TIF) [file pone.0022023.s001.tif]

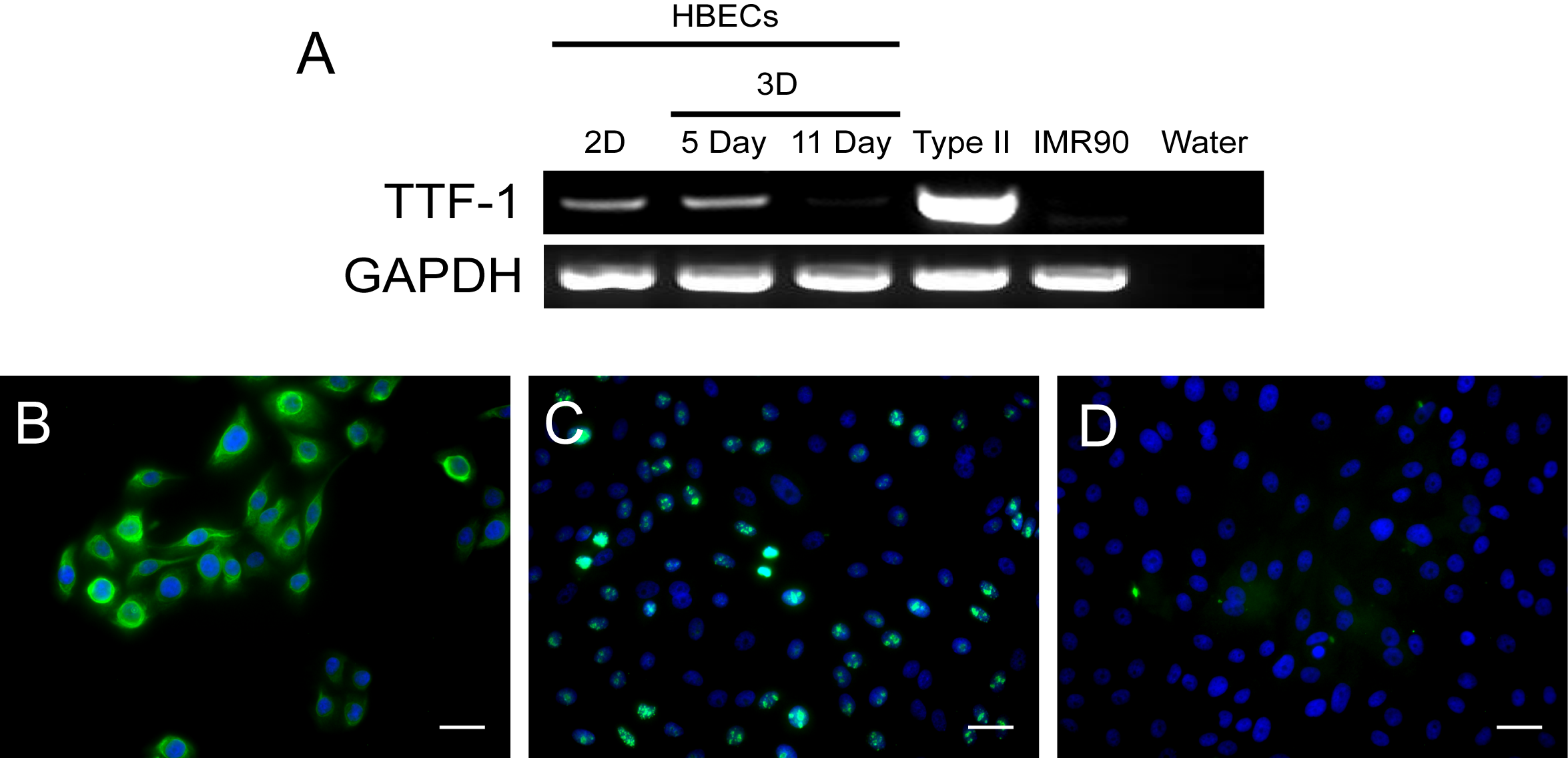

Supplement: Figure S2 — TTF1 Expression in HBEC3 KT Cell Line. (A) RT-PCR analysis of TTF-1 in HBEC3 KTs cultured under differentiation conditions in 2D or within Matrigel™ compared to human fetal Type II pneumocytes and IMR90 fibroblasts. (B–D) Immunofluorescence staining of TTF-1 in HBEC3 KT cells 2 days (B), 5 days (C), and 7 days (D) after transition from logarithmic to differentiation conditions in 2D. Scale bars 20 µm. (TIF) [file pone.0022023.s002.tif]
