# Supplementary material for: Multipotent Capacity of Immortalized Human Bronchial Epithelial Cells
Source: PLoS One. 2011 Jul 7;6(7):e22023. doi: 10.1371/journal.pone.0022023 (PMC3131301; doi:10.1371/journal.pone.0022023)
Supplement: Table S1 — TaqMan® Gene Expression Assay IDs. TaqMan® Gene Expression Assay IDs and for expression analysis reported in Figure 2. (DOC) [file pone.0022023.s003.doc]

| **Gene** | **RefSeq ID** | **Pathway** | **TaqMan® Assay ID** |
| --- | --- | --- | --- |
| PTCH1 | NM_000264 | Hedgehog | Hs00181117_m1 |
| PTCH2 | NM_003738.3 | Hedgehog | Hs00184804_m1 |
| SMO | NM_005631 | Hedgehog | Hs00170665_m1 |
| GLI1 | NM_005269 | Hedgehog | Hs00171790_m1 |
| GLI3 | NM_000168 | Hedgehog | Hs00609233_m1 |
| DLL1 | NM_005618 | Notch | Hs00194509_m1 |
| DLL4 | NM_019074 | Notch | Hs00184092_m1 |
| JAG1 | NM_000214 | Notch | Hs00164982_m1 |
| JAG2 | NM_002226 and NM_145159 | Notch | Hs00171432_m1 |
| NOTCH1 | NM_017617 | Notch | Hs00413187_m1 |
| NOTCH2 | NM_024408 | Notch | Hs00225747_m1 |
| NOTCH3 | NM_000435 | Notch | Hs00166432_m1 |
| HES1 | NM_005524 | Notch | Hs00172878_m1 |
| HEY1 | NM_012258 and NM_001040708 | Notch | Hs00232618_m1 |
| HEY2 | NM_012259 | Notch | Hs00232622_m1 |
| MAML2 | NM_032427.1 | Notch | Hs00287205_m1 |
| FZD1 | NM_003505 | Wnt | Hs00268943_s1 |
| DVL2 | NM_004422.2 | Wnt | Hs00182901_m1 |
| DVL3 | NM_004423.3 | Wnt | Hs00610263_m1 |
| APC | NM_000038 | Wnt | Hs00181051_m1 |
| CTNNB | NM_001904 | Wnt | Hs00170025_m1 |
| OCT4 | NM_002701 | Other (stem cell) | Hs01895061_u1 |
| NANOG | NM_024865 | Other (stem cell) | Hs02387400_g1 |
| SOX2 | NM_003106 | Other (stem cell) | Hs00602736_s1 |
| MYC | NM_002467 | Other (stem cell) | Hs00153408_m1 |
| BMI1 | NM_005180 | Other (stem cell) | Hs00180411_m1 |
| KLF4 | NM_004235 | Other (stem cell) | Hs00358836_m1 |
| GAPDH | NM_002046 |  | 4352934E |

**Table S1. TaqMan® Gene Expression Assay IDs.**
